# Proteome-wide analysis of lysine β-hydroxybutyrylation in the myocardium of diabetic rat model with cardiomyopathy

**DOI:** 10.3389/fcvm.2022.1066822

**Published:** 2023-01-09

**Authors:** Weiguang Luo, Mei He, Qizhi Luo, Yi Li

**Affiliations:** ^1^Department of Clinical Laboratory, Henan Provincial People’s Hospital, People’s Hospital of Zhengzhou University, Zhengzhou, Henan, China; ^2^Henan Medical Key Laboratory of Arrhythmia, The 7th People’s Hospital of Zhengzhou, Zhengzhou Cardiovascular Hospital, Zhengzhou, China; ^3^Department of Immunology, Basic Medical School of Central South University, Changsha, Hunan, China; ^4^Department of Clinical Laboratory, Henan Provincial People’s Hospital, Henan University People’s Hospital, Zhengzhou, Henan, China

**Keywords:** lysine β-hydroxybutyrylation, post-translational modification, proteomics, liquid chromatography-mass spectrometry, diabetic cardiomyopathy

## Abstract

Lysine ß-hydroxybutyrylation (kbhb), a novel modification of lysine residues with the ß-hydroxybuty group, is associated with ketone metabolism in numerous species. However, its potential role in diabetes, especially in diabetic cardiomyopathy (DCM), remains largely unexplored. In this study, using affinity enrichment and liquid chromatography-mass spectrometry (LC-MS/MS) method, we quantitatively analyze the kbhb residues on heart tissues of a DCM model rat. A total of 3,520 kbhb sites in 1,089 proteins were identified in this study. Further analysis showed that 336 kbhb sites in 143 proteins were differentially expressed between the heart tissues of DCM and wild-type rats. Among them, 284 kbhb sites in 96 proteins were upregulated, while 52 kbhb sites in 47 proteins were downregulated. Bioinformatic analysis of the proteomic results revealed that these kbhb-modified proteins were widely distributed in various components and involved in a wide range of cellular functions and biological processes (BPs). Functional analysis showed that the kbhb-modified proteins were involved in the tricarboxylic acid cycle, oxidative phosphorylation, and propanoate metabolism. Our findings demonstrated how kbhb is related to many metabolic pathways and is mainly involved in energy metabolism. These results provide the first global investigation of the kbhb profile in DCM progression and can be an essential resource to explore DCM’s pathogenesis further.

## 1. Introduction

Diabetic cardiomyopathy (DCM) is characterized by diabetes-associated alterations in the structure and function of the myocardium, independently of coronary artery diseases or hypertension. Almost 8% of mortality in patients with diabetes is caused by DCM (1). A critical step toward improving DCM treatment and mortality involves investigating the underlying biological mechanisms leading to the initiation and progression of DCM. Biological DCM-associated abnormalities include hyperglycemia, mitochondrial dysfunction, extra oxidative stress, abnormal insulin response, cardiac inflammation, impaired glucose metabolism, changes to the extracellular matrix’s composition, and accelerated cardiac fibrosis (2–4). However, the molecular mechanisms underlying DCM remain to be further explored.

Post-translational modifications (PTMs) are a cluster of reversible protein modifications influencing the properties and functions of a protein (5). Since PTMs affect a protein’s charge, hydrophobicity, stability, and activity, they have been recognized to involve in modulating multiple intracellular metabolisms (6, 7). Numerous pieces of research have demonstrated that various histone PTMs dynamically regulate chromatin structure and function and affect gene expression (8, 9). Lysine is a frequent target for PTM. To date, the discovered lysine PTMs include phosphorylation, acetylation, ubiquitination, butyrylation, propionylation, 2-hydroxyisobutyrylation, succinylation, malonylation, crotonylation, glutarylation, and β-hydroxybutyrylation (10, 11).

Lysine ß-hydroxybutyrylation (kbhb) is a novel histone PTM involving the transfer of a ß-hydroxybutyric group to a lysine residue (12). kbhb of histone was first discovered in cells treated with β-hydroxybutyrate (βOHB) and in the livers of mice under the conditions of long-term fasting or diabetic ketoacidosis. Kbhb of histone has been identified in yeast, flies, mouse, and human cells. H3K9 kbhb is increased in active gene promoters, which are associated with upregulated genes in a starvation-responsive manner, indicating its crucial role in regulating starvation-responsive metabolism and epigenetics (12). The p53 kbhb reduced its acetylation and downstream activation of the genes p21 and PUMA, undermining the p53-associated apoptosis and cell growth arrest (13).

A recent study indicated that the H3K9 kbhb enhanced the expression of matrix metalloproteinases-2 (MMP2) of the zinc-dependent endopeptidases family that degrades extracellular matrix, antagonizing glomerulosclerosis in a diabetic rat model (14). It is also reported that H3K9 kbhb enhances VEGF gene expression, attenuating endothelium injury in diabetic rats. Terranova et al. (15) demonstrated that H3K9 kbhb is related to active chromatin states and plays a vital role in the expression of metabolic genes (16). These findings illustrated that kbhb plays a vital role in maintaining cellular physiology.

Few studies have reported the global analysis of kbhb proteins in disease progression. The comprehensive kbhb protein expression, alterations, regulatory enzymes, and substrates have been poorly explored, hindering the understanding of the underlying mechanisms of this modification in biological progress. PTMs such as O-GlcNAcylation and scalation have been proven to be associated with the progression of DCM (17, 18). In this study, we aimed to investigate the quantitative global proteome of kbhb in related proteins to provide a comprehensive view of the molecular mechanisms underlying DCM.

## 2. Materials and methods

### 2.1. Animal model and treatment

A total of 10 male healthy Sprague-Dawley (SD) rats (6–8 weeks, 140 ± 10 g), which were provided by the Experimental Animal Center, were housed in individually ventilated cages at standard laboratory conditions (temperature: 22–25°C, humidity: 50–60%) with a 12-h light/dark cycle. After 7 days of adaptation, the rats were randomly divided into a control group and a DCM group. The rats in the DCM group were treated with a high-sugar and high-fat diet, as reported previously (19, 20). The rats in the DCM group were injected with a single dose of streptozotocin (STZ) (60 mg/kg, Sigma-Aldrich) to induce diabetes 8 weeks later. The control group performed the same simulated injection operation. One week after the STZ injection, the blood was collected from the tail vein, and the fasting blood glucose (FBG) levels and the βOHB were measured by a glucose and ketones meter (Abbott, USA) using the test strip for glucose or βOHB, respectively. Rats with an FBG > 12 mmol/L were considered diabetes mellitus (21).

### 2.2. Oral glucose tolerance test (OGTT) and echocardiography

The oral glucose tolerance test (OGTT) is used to diagnose diabetes. After a 12-h overnight fast, rats were fed 50% glucose at a dose of 2 g/kg of body weight, and the blood glucose levels were assessed at 0, 30, 60, and 120 min, respectively.

The rats were anesthetized by chloral hydrate (0.4 ml/100 g), and their cardiac structure and function were assessed using two-dimensional echocardiography (VisualSonics, Toronto, Canada). The left ventricular end-diastolic diameter (LVEDD), left ventricular end-systolic diameter (LVESD), left fractional ventricular shortening (LVFS), and left ventricular ejection fraction (LVEF) were measured.

### 2.3. Histological analysis

Histological analysis was performed using hematoxylin and eosin (H&E) to detect structural abnormalities. Masson’s trichrome staining was used for evaluating fibrosis. Ventricular myocardial tissue from the heart of rats was fixed and paraffin-embedded, then was sliced into 5 μm sections. H&E staining and mason staining were performed according to the instructions. The sections were analyzed under a light microscope (Automated Fluorescence Microscope; Olympus, Tokyo, Japan). ß-hydroxybutyrylation proteins in myocardial tissue were detected by immunohistochemistry. Briefly, the sections were incubated overnight with primary antibodies against βOHB lysine (PTM-1201RM, Jingjie PTM, China) at 4°C and then were incubated with secondary antibodies.

### 2.4. Coomassie brilliant blue staining and western blotting analysis

Supernatants from the total fractions were mixed with loading buffer and boiled for 10 min. First, the same amount of protein (20 μg) of each sample was separated on an SDS-PAGE gel. For Coomassie brilliant blue staining, the gel was submerged in the staining solution (Solarbio, Beijing, China) for 2 h with slow shaking. The gel was then destained in a solution of 5% acetic acid overnight and stored in 20% glycerin for further analysis. The proteins were separated on the SDS-PAGE gel for western blotting analysis and then transferred to nitrocellulose membranes. They were then incubated with 5% non-fat milk at room temperature for 1 h, washed, and incubated in the primary antibodies against β-hydroxybutyrylation lysine (PTM-1201RM, Jingjie PTM, Hangzhou, China). Membranes were then rewashed and incubated with the secondary antibody for another 2 h at room temperature with shaking. All antibodies were diluted by washing in TBST buffer. Membranes were developed using a chemiluminescence ECL kit (WBKLS0500, Millipore, Darmstadt, Germany), and all images were analyzed using a ChemiDoc Touch Imaging System (Bio-Rad, CA, USA).

### 2.5. Protein extraction

The samples were ground into a powder by snap-freezing in liquid nitrogen. Afterward, a lysis buffer containing 8 M urea, 0.1% protease inhibitor cocktail, 3 μM trichostatin A (TSA), and 0.05 M nicotinamide (NAM) was added to the cell powder in a 4:1 ratio, and then sonicated on ice using a high-intensity sonicator (Scientz). The remaining debris was then removed by centrifugation at 12,000 × *g* for 10 min at 4°C. Finally, the supernatant was collected, and the protein concentration was determined with the BCA kit according to the manufacturer’s instructions.

### 2.6. Trypsin digestion

Equal amounts of the protein prepared in the previous Section “2.5 Protein extraction” were taken and adjusted to the same volume with lysis buffer. The same volume of 40% tricarboxylic acid was added to the supernatant. The mixture was then vortexed and left at 4°C for 2 h. Then, the supernatant was discarded by centrifuging at 4,500 × *g* at 4°C for 5 min. The protein pellet obtained was washed three times with ice-cold acetone and redissolved in a buffer containing 20% tetraethylammonium bromide. Later, the protein was sonicated using a sonicator. The protein solution was reduced using 5 mM dithiothreitol at 56°C for 30 min and then alkylated with 11 mM iodoacetamide at room temperature for 15 min in the dark. Trypsin digestion was carried out as previously described (22, 23). Briefly, trypsin was added into the sample solution prepared earlier at a mass ratio of 1:50 (enzyme/protein) for digesting overnight at room temperature. Second digestion was carried out at a mass ratio of 1:100 (enzyme/protein) for 4 h. Finally, the peptides were desalted by passing through a C18 SPE column.

### 2.7. Affinity enrichment

The peptides obtained from the digestion in the previous step were dissolved in the NETN buffer (100 mM NaCl, 1 mM EDTA, 50 mM Tris–HCl, and 0.5% NP-40). Then, the abovementioned mixture was incubated with pre-washed resin and conjugated with anti-β-hydroxybutyrylated lysine antibody (Cat. No. 1204, PTM Biolabs, Hangzhou, China) by gentle shaking for 12 h at 4°C (24). Then, the resin was washed four times with NETN buffer and then washed two times with deionized water. In the end, the resin-bound peptides were eluted with 0.1% trifluoroacetic acid by washing three times. The eluate obtained was collected and vacuum-dried. The resulting peptides were desalinated according to the instructions of C18 ZipTips (Millipore, Billerica, MA), followed by liquid chromatography-mass spectrometry (LC-MS/MS) analysis (25).

### 2.8. Liquid chromatography-mass spectrometry (LC-MS/MS) analysis

Peptides were dissolved in solvent A [0.1% formic acid (FA) and 2% acetonitrile] and separated using EASY-nLC 1200 ultra-performance liquid chromatography (UPLC). The gradient contained an increase from 9 to 25% solvent B (0.1% FA in 90% acetonitrile) for 36 min, 25 to 35% for 18 min, 80% in 4 min, then holding at 80% for the last 4 min, all at a constant flow rate of 500 ml/min on an EASY-nLC 1200 UPLC system (22).

The peptides obtained earlier were subjected to nanospray ionization and analyzed by Q Exactive™ HF-X mass spectrometer. The electrospray voltage was set to 2.1 kV. The scan resolution was set to 120,000 with a scan range of 350–1,600 m/z, where the data acquisition mode uses the data-dependent scanning (DDA) program. A total of 20 most abundant peptide precursors were then selected for further MS/MS analysis with 30-s dynamic exclusion. The normalized collision energy (NCE) was set as 28%. The resulting fragments were detected in the Orbitrap at a resolution of 15,000. The fixed first mass was set as 100 m/z. The automatic gain control (AGC) target was set at 1E5, with an intensity threshold of 5E4, and a maximum injection time of 100 ms (26).

### 2.9. Database search

The MaxQuant software was used to process the obtained MS/MS data with the integrated Andromeda search engine (version 1.6.15.0). The resulting MS/MS data were searched against the UniProt Rattus norvegicus (29,923 sequences) database concatenated with the reverse decoy database. The cleavage enzyme was Trypsin/P, and four missed cleavages were allowed (22).

The mass error of the precursor ions in the first search and main search was set to 20 ppm and 4.5 ppm, respectively, and the mass error of the fragment ions was 20 ppm. Carbamidomethylation on Cys was specified as fixed modification and oxidation on Met, ß-hydroxybutyrylation on lysine, acetylation on lysine, and acetylation on protein *N*-terminal were specified as variable modifications. False discovery rate (FDR) thresholds for peptide and modification sites were set at 1% (27). Seven amino acid residues were defined as the minimum length of the peptide. The other parameters in MaxQuant were assigned to the default values. The localization probability of ß-hydroxybutyrylation sites was set as 0.75 (22).

### 2.10. Data analysis

The quantitative values for each protein in all the samples were normalized according to their average abundance in a single sample. Moreover, the ratio of the mean relative quantitative values of modified sites in the DCM and control groups was taken as the fold change (FC) for selecting different modified sites. The quantitative data were used to compare the modified sites and protein abundance between DCM and the control groups. A *t*-test was performed using the standardized and log_2_ transformed values. The *p*-value < 0.05 was considered significant. The intensities of proteins quantified in all samples were subjected to log_2_ transformation and then the principal component analysis (PCA) was performed using Perseus version 1.6.1.2 (28).

### 2.11. Bioinformatics analysis

Gene ontology (GO) analysis of the resulting β-hydroxybutyrylation proteome was derived from the UniProt-GOA database^1^ (29). Then, proteins were classified by GO annotation based on three categories, namely, biological process (BP), cellular component (CC), and molecular function (MF). The subcellular localization of the protein obtained by mass spectrometry was predicted using Wolfpsort (30). Secondary structures of proteins were predicted by NetSurfP (31). The MOMO software^2^, based on motif-x (32), was employed to derive the sequence model around each modified site in all protein sequences (10 amino acids upstream and downstream of the β-hydroxybutyrylated site). The results of the Kyoto Encyclopedia of Genes and Genomes (KEGG) pathway analysis were obtained from the DAVID^3^. For GO and KEGG functional enrichment, a two-tailed Fisher’s exact test was used to test the enrichment of the differentially expressed protein across all the identified proteins. A corrected *p*-value < 0.05 is considered statistically significant for GO and KEGG pathway analyses. The physical and functional interaction network of all differentially expressed proteins was constructed using the STRING database (version 11.5^4^) and was visualized by Cytoscape (version 3.9.1^5^).

## 3. Results

### 3.1. Establishment of a DCM rat model

**FIGURE 1:**
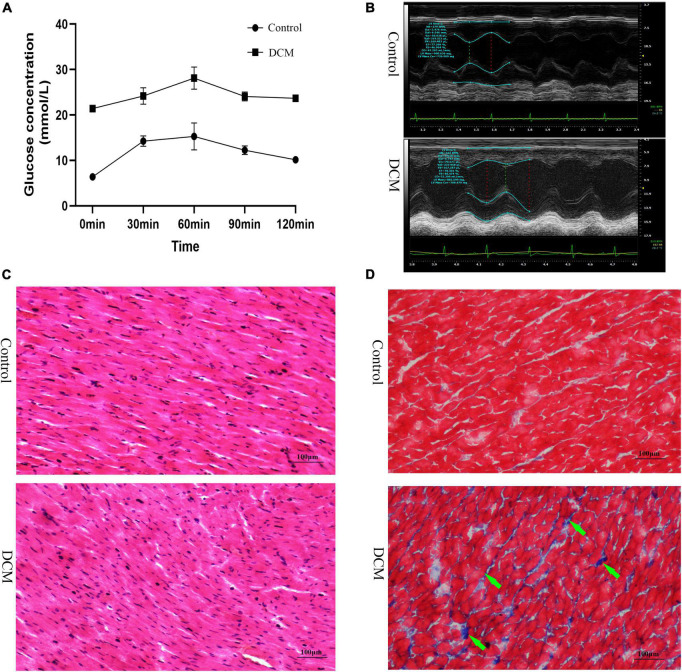
Establishment of diabetic cardiomyopathy (DCM) rat modes. **(A)** The results of the oral glucose tolerance test (OGTT) (*n* = 3 rats per group). **(B)** Representative M mode tracing of echocardiographs (*n* = 3 rats per group). **(C)** Hematoxylin and eosin (H&E) staining of specimens from the control and DM groups (*n* = 3 rats per group, scale bar = 100 μm). **(D)** Masson staining specimens from the control and DM groups (*n* = 3 rats per group, scale bar = 100 μm). The green arrows represent the blue collagen fibers.

First, we performed the proteome analysis of β-hydroxybutyrylated proteins and their modified residues in the heart of diabetic rat models to investigate the role of this phenomenon in the development of DCM. The experimental flow of this research is shown in Supplementary Figure 1. The FBG and the blood glucose concentrations of OGTT in the DCM group were more than 20 mmol/L (Figure 1A and Supplementary Figure 2A), indicating the successful onset of diabetes mellitus. In contrast to the control group, the diabetic group exhibited increased LVESD, LVEDD, and reduced LVEF (Figure 1B and Supplementary Figures 2B–D). H&E staining revealed intact cardiomyocytes in the control group, with uniform dispersion and an ordered organization. The cytoplasm of myocardial cells was uniformly stained, and the nuclei were in the center of the cytoplasm, with similar size and oval or round shape (Figure 1C). In the DCM group, myocardial cells were lighter in color, had diverse sizes, and were disordered and organized. In contrast, cytoplasmic staining was uneven and dispersed, with some of the cardiomyocytes being fragmented or out of shape. Masson’s trichome staining revealed homogeneous, uniformly red-stained myocardial cells in the control group, with just a few blue fibrous structures visible (Figure 1C). However, many blue collagen fibers exist in the DCM group’s intercellular spaces between red myocardial tissues (Figure 1D). These results demonstrate that DCM develops in the heart of diabetic rats.

### 3.2. Proteome-scale identification of lysine β-hydroxybutyrylation sites

**FIGURE 2:**
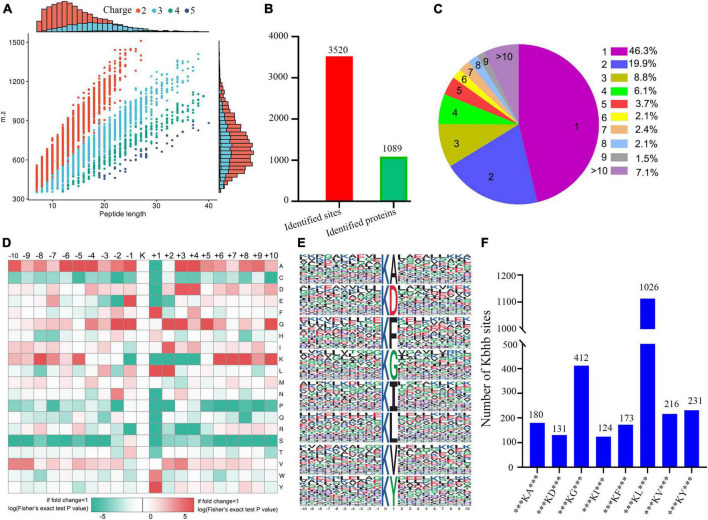
Properties of the lysine β-hydroxybutyrylation (kbhb) sites. **(A)** Distributions of peptide length for all the β-hydroxybutyrylation sites identified in the rats (*n* = 3 rats per group). **(B)** The number of identified sites and proteins. **(C)** A pie chart of the number and percentage of β-hydroxybutyrylation residues per protein. **(D)** A heat map of the ± 10 amino acid compositional frequencies surrounding the β-hydroxybutyrylation residues. **(E)** The modified peptide motifs containing ± 10 amino acids surrounding the identified residues. **(F)** The number of β-hydroxybutyrylation sequence motifs.

The concentration of βOHB in the blood was higher (Supplementary Figure 3) in the DCM group compared to the controls. We performed western blotting before the proteomic experiments to detect the abundance of β-hydroxybutyrylated proteins in the heart tissue of rats, which showed their higher abundance in the myocardial tissue of the DCM group mice (Supplementary Figure 4). Immunohistochemical staining also showed a significant increase in the expression of β-hydroxybutyrylated proteins in the DCM group (Supplementary Figure 5). Subsequently, we performed the global β-hydroxybutyrylation analysis using affinity enrichment followed by high-resolution LC-MS/MS. Errors in the quality of the reads and the distribution of all identified peptides were detected to validate the mass spectrometry results. As shown in Supplementary Figure 6, all the peptide errors were less than 5 ppm, and distributed near to zero, indicating the accuracy and reliability of the dataset obtained in this study. The amino acids of most peptides were distributed between 7 and 20 (Figure 2A), indicating that the processed samples matched the requirements for proteomics analysis.

A total of 3,520 β-hydroxybutyrylated lysine sites in 1,089 proteins were identified in this study (Figure 2B; Additional file 2: Supplementary Table 1). We found that 46.3% (484 out of 1,089) of the identified proteins had one kbhb site (Figure 2C). The percentages of proteins with two, three, four, and five lysine β-hydroxybutyrylated residues were 19.9% (209 out of 1,089), 8.8% (92 out of 1,089), 6.1% (64 out of 1,089), and 3.7% (39 out of 1,089), respectively. Interestingly, 7.1% (74 out of 1,089) of the peptides were modified at more than 10 lysine residues. To further investigate this study’s kbhb features, we used the MoMo software and hierarchical clustering to analyze the flanking sequences from −10 to +10 of the kbhb site. Our analysis revealed that alanine (A), glycine (G), lysine (K), leucine (L), and valine (V) had the highest frequency, while proline (P) and serine (S) had the lowest frequency (Figure 2D). The frequency of alanine (A) residue at positions −6 to −4 and +3 to +4 was the highest. The positively charged lysine (K) residue was enriched at positions −8 to −5 and +6 to +10, not at positions +1 to +4. The amino acid sequences of kbhb peptides were further analyzed by the Motif-X program (Figure 2E). Consistent with the results of the amino acid heat map (Figure 2D), eight conserved motifs, ***KbhbA***, ***KbhbD***, ***KbhbG***, ***KbhbI***, ***KbhbF***, ***Kbhbl***, ***KbhbV***, and ***KbhbY*** (kbhb represents the modified lysine site and * represents a random amino acid site), were obtained. These conserved sequences matched 2,493 identified modified peptides, showing varying abundances (Figure 2F and Additional File 2: Supplementary Table 2).

### 3.3. Quantitative signatures of altered lysine β-hydroxybutyrylation peptides

**FIGURE 3:**
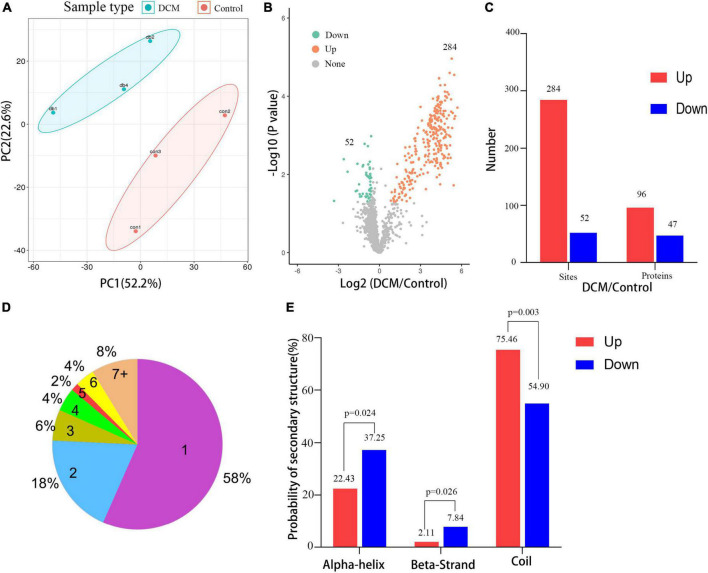
Quantitative signatures of different lysine β-hydroxybutyrylation (kbhb) sites in heart tissue of diabetic cardiomyopathy (DCM) rats. **(A)** Principal component analysis (PCA) was performed based on the log_2_-transformed intensity of proteins identified in the three replicates (*n* = 3 rats per group). **(B)** Volcano plot graphs representing the comparative analysis between the DM group and control group. The X-axes correspond to the ratio of intensities in the log_2_ scale for each protein in the two samples, and the Y-axes are the *p*-value in the log_10_ scale. Blue dots represent proteins downregulated in DM rats, and orange dots correspond to proteins upregulated in DM rats. **(C)** The number of lysine β-hydroxybutyrylation (kbhb) sites and proteins. **(D)** The number and percentage of β-hydroxybutyrylation residues per protein. **(E)** Analysis of the secondary structure of the β-hydroxybutyrylated protein.

Next, a quantitative analysis of the kbhb proteomic data was performed to check whether the tissue samples reflected the difference between normal and DCM rats. A total of 2,295 kbhb sites were subjected to the following quantitative analysis. PCA was done to classify the kbhb proteomic results of the normal tissues (pink area) and DCM tissues (blue area) (Figure 3A). The kbhb sites with FC ratios of >1.5 for the upregulation and <0.67 for the downregulation threshold and a *p*-value < 0.05 were considered to be significantly differentially expressed between DCM and normal tissues and, thereby, selected for further analysis. Compared to the control group, 284 kbhb sites in 96 proteins were increased in DCM heart tissues (blue) and 52 kbhb areas in 47 proteins, which were decreased in DCM heart tissues (pink) (Figures 3B, C, Additional file 2: Supplementary Table 3). Further analysis showed that about 58% (77 out of 136) of the identified kbhb proteins were modified at one lysine residue, 18% (26 out of 136) were altered at two lysine residues, and 34% (33 out of 136) were modified at more than two lysine residues (Figure 3D). Moreover, we analyzed the secondary structures of all the kbhb proteins in DCM rats to investigate the relationship between the protein secondary structures and the modified lysine residues. The results showed that the upregulated kbhb sites were more frequently found in *Escherichia coli* (*p* = 0.003), while the downregulated kbhb sites were more commonly found in the alpha helix (*p* = 0.024) and beta strand (*p* = 0.026) (Figure 3E).

### 3.4. Functional classification and subcellular localization of altered kbhb proteins in control and DCM heart

**FIGURE 4:**
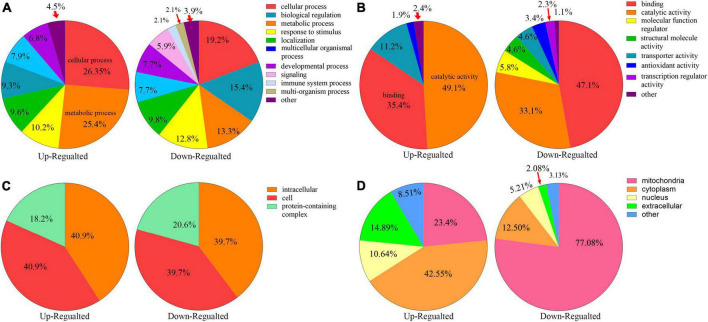
Functional classifications of different lysine β-hydroxybutyrylation (kbhb) proteins in heart tissue of diabetic cardiomyopathy (DCM) rats. **(A)** Classification of kbhb proteins according to their biological processes (BPs). **(B)** Classification of kbhb proteins based on their molecular functions (MF). **(C)** Classification of kbhb proteins according to their cellular components (CC). **(D)** Analysis of the subcellular localization of the kbhb proteins.

All the differentially expressed proteins were functionally classified using GO and categorized into BPs, CCs, and MFs (Figures 4A–C, Additional file 2: Supplementary Table 4) to further study the potential roles of kbhb proteins in DCM. In BP, many upregulated kbhb proteins were involved in cellular processes (26.4%), metabolic processes (25.4%), and response to stimulus (10.2%) in DCM heart tissues. In comparison, the downregulated kbhb proteins were mainly related to the cellular process (19.2%), biological regulation (15.4%), and metabolic process (13.3%) (Figure 4A). MF analysis revealed that the upregulated kbhb proteins were mainly involved in catalytic activity (49.1%). In contrast, the downregulated kbhb proteins were primarily associated with binding (47.1%) (Figure 4B), with no difference between upregulated and downregulated kbhb proteins in the CC category (Figure 4C). These results suggested that kbhb might alter the biological function of proteins, further influencing various BPs. As shown in Figure 4D (additional file 2: Supplementary Table 5), the analysis of subcellular localization of altered kbhb proteins demonstrated that the changed proteins were widely distributed in various components in the cell. The upregulated kbhb proteins were mainly located in the cytoplasm (42.55%), while the downregulated ones were primarily distributed in mitochondria (77.08%).

### 3.5. Functional enrichment analysis of altered kbhb proteins in DCM

**FIGURE 5:**
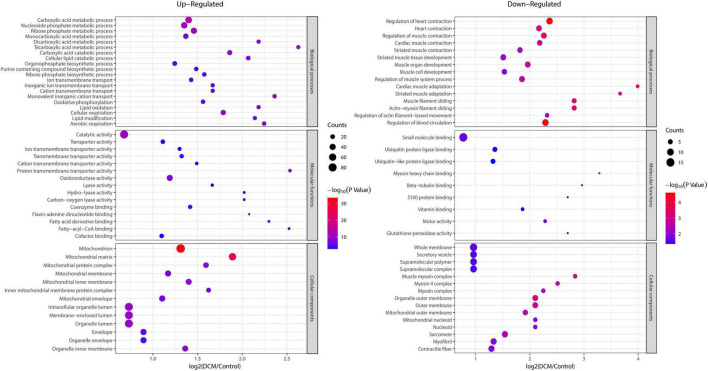
Enrichment analysis of the lysine β-hydroxybutyrylation (kbhb) proteins based on gene ontology (GO) in heart tissue of diabetic cardiomyopathy (DCM) rats. **(left)** Upregulated. **(right)** Downregulated.

**FIGURE 6:**
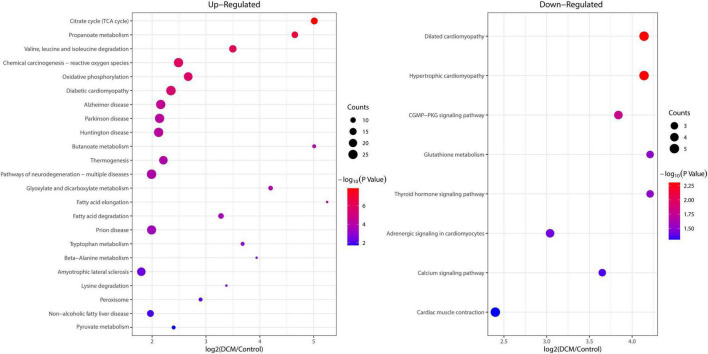
Enrichment analysis of the lysine β-hydroxybutyrylation (kbhb) proteins in heart tissue of diabetic cardiomyopathy (DCM) rats according to the Kyoto Encyclopedia of Genes and Genomes (KEGG) pathway. **(left)** Upregulated. **(right)** Downregulated.

To reveal the preferred target protein types of kbhb proteins, we performed a functional enrichment analysis of the identified kbhb proteins *via* GO and KEGG pathway analyses. In the BP analysis, we observed that upregulated proteins were enriched in the metabolic, catabolic, and biosynthetic processes. In contrast, most downregulated proteins were enriched in heart and muscle contraction. Among MF, the upregulated proteins were highly enriched in catalytic activity, oxidoreductase activity, and transmembrane transporter activity. In contrast, the downregulated proteins were enriched in small molecule binding and protein ligase binding. In the enrichment analysis of CC, the upregulated proteins mainly exhibited mitochondrial and organelle localization. In contrast, the downregulated proteins were enriched in cytoplasms such as secretory vesicles and supramolecular complex (Figure 5 and Additional file 2: Supplementary Table 6). The KEGG pathway analysis indicated that the citrate cycle (TCA cycle), propanoate metabolism, and oxidative phosphorylation were enriched among the upregulated kbhb proteins. Meanwhile, upregulated kbhb proteins were also involved in developing DCM. In contrast, the pathways enriched among the downregulated proteins were the cGMP-PKG signaling pathway and glutathione metabolism. In addition, the downregulated proteins were related to the process of dilated cardiomyopathy and hypertrophic cardiomyopathy. (Figure 6 and Additional file 2: Supplementary Table 7).

### 3.6. Protein–protein interactions network of altered kbhb proteins in DCM heart

**FIGURE 7:**
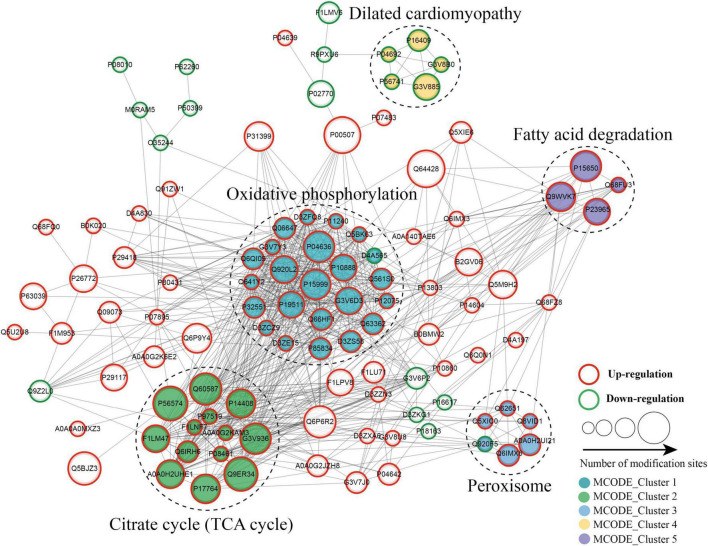
Protein–protein interaction (PPI) networks of lysine β-hydroxybutyrylation (kbhb) proteins in heart tissue of diabetic cardiomyopathy (DCM) rats.

The protein–protein interaction (PPI) network analysis was performed to identify the chief nodes and the significant BPs among the differentially expressed kbhb proteins. As shown in Figure 7 (Additional file 2: Supplementary Table 8), 107 kbhb states and 665 interactions were mapped in the PPI network database, exhibiting a global view of the pathways regulated by differentially expressed kbhb proteins. Cytoscape software and Minimal Common Oncology Data Elements (MCODE) analysis revealed a few highly associated sub-networks of kbhb proteins. Consistent with the enrichment results of KEGG, the kbhb proteins related to oxidative phosphorylation, citrate cycle (TCA cycles), peroxisome, and fatty acid degradation were upregulated. In contrast, several kbhb proteins related to dilated cardiomyopathy were downregulated in DCM rats (Figure 7).

## 4. Discussion

Diabetic cardiomyopathy, a severe cardiovascular complication from diabetes, causes structural and functional abnormalities of the myocardium, eventually leading to heart failure. Several molecular and cellular mechanisms are involved in the development of DCM (33). Recently, PTMs such as O-GlcNAcylation and succinylation have been associated with the progression of DCM (17, 18). Interestingly, ketone bodies partially replace glucose as an energy source in the diabetic heart (34). βOHB accounts for 70% of ketone bodies and is the most abundant in circulating ketones (35). βOHB not only serves as an alternative energy supply to the heart but also has various kinds of signaling functions, including gene expression and regulation (36). βOHB induced the histone lysine kbhb, which regulates gene transcription. The purpose of this study was to investigate the quantitative global proteome of kbhb in related proteins, providing a comprehensive view of the molecular mechanisms underlying DCM.

We established a DCM rat model with a high-sugar and high-fat diet combined with low-dose STZ intraperitoneal injection. The OGTT results showed that the FBG and blood glucose at 2 h were more significant than 20 mmol/L, indicating the successful onset of diabetes mellitus. In this study, the weight of DCM rats reduces significantly. Due to abnormal energy metabolism in diabetes, the degradation of protein increases, resulting in the consumption of muscle protein and eventually inducing muscle atrophy and weight loss (37). DCM is characterized by systolic and diastolic dysfunction. In line with a previous report (38), this study corroborated how impaired heart function of rats was manifested by increased LVESD, LVEDD, and reduced LVEF. In addition, the H&E and Masson’s staining showed aberrantly sized and disorderly arranged cardiomyocytes and increased collagen deposition in the DCM heart tissues, consistent with previous reports (19, 39).

We then carried out a quantitative proteomics analysis to explore the role of kbhb proteins in DCM progression. A total of 3,520 lysine sites in 1,089 proteins identified in this study were β-hydroxybutyrylated–further analysis showed that 336 kbhb sites in 143 proteins were differentially expressed in DCM tissues. Among them, 284 kbhb sites in 96 proteins increased, while 52 kbhb sites in 47 proteins decreased in DCM tissues. The data showed the upregulated proteins in DCM tissues to be enriched in the TCA cycle, oxidative phosphorylation, and propanoate metabolism. In contrast, the downregulated proteins were enriched in the dilated cardiomyopathy, cGMP-PKG signaling pathway, and glutathione metabolism. A study on the STZ-induced type I DM mice model detected a significant increase of global protein kbhb in the liver and kidney but not in the heart (40). However, the present study found increased kbhb protein levels in the DCM heart tissues and demonstrated them to be related to DCM progression.

Kbhb is formed by amide bonding with the βOHB carboxyl group on the ε amino group of protein lysine (41). Both *in vivo* and *in vitro* investigations have indicated that elevated βOHB increased the level of kbhb (40, 42). Consistent with special reports, our results showed a significantly increased blood concentration of βOHB in DM rats. The possible reason is that in the case of a high-sugar and high-fat diet, abnormal energy metabolism of rats causes the liver to produce a large amount of fatty acid-derived βOHB, leading to elevated levels in the blood (43). As “super fuels” for the heart, ketones generate more ATP than fatty acids and glucose (44). Therefore, the increased level of βOHB in the blood and the uptake of βOHB from the cardiomyocyte in diabetes might be an adaptive mechanism in order to improve cardiac function.

Under normal conditions, fatty acids and glucose are the primary energy sources for the heart to maintain continuous systolic movement to support cardiac pumping. However, during aberrant metabolic conditions such as diabetes and insulin resistance, glucose utilization by cardiomyocytes decreases, while the utilization of fatty acids and ketone bodies increases (45). Cardiomyocytes need to adapt to these energy transitions to maintain myocardial energy metabolism. The consumption βOHB of the heart significantly improved cardiac function and the stabilization of membrane potential, leading to the enhanced antiarrhythmic potential of cardiomyocytes (46). Elevated βOHB ameliorated heart aortic endothelial injury through increasing kbhb on histone, inducing VEGF expression (15). Ketone bodies produced by the body can fully enter the energy metabolism to generate ATP, but in the case of uncontrolled hyperglycemia and high fatty acids, it was affected by the utilization of ketone bodies by cardiomyocytes. The increased intermediate metabolite produces more βOHB carboxyl, which makes the protein kbhb in the cell. In this study, the altered kbhb protein was mainly enriched in the oxidative phosphorylation and the tricarboxylic acid cycle, which confirmed that extra ketone body intermediate metabolites were produced. The results in this study suggest that βOHB may influence the pathology of DCM by increasing non-histone kbhb. The level of kbhb may be a biomarker to monitor ketones metabolism, providing a new way to assess the effects of ketones therapy.

Aberrant energy metabolism is an essential mechanism for the development of DCM (47). The results of this study indicated that the upregulated kbhb proteins in DCM tissues were mainly enriched in the TCA cycle and oxidative phosphorylation (Figures 6, 7). At the same time, the mitochondria use both fatty acids and glucose to produce ATP *via* the TCA cycle and oxidative phosphorylation. A comprehensive analysis of kbhb proteins from starved mouse liver also revealed that kbhb-modified proteins were enriched in fatty acid (lipid and acyl-CoA metabolic processes, β-oxidation), TCA cycle, and ATP metabolic processes (40). kbhb seems to be involved in regulating ATP synthesis and contributing to the development of DCM. Future studies are needed to reveal the underlying mechanism.

Oxidative phosphorylation provides 95% of the ATP needed to maintain cardiac function and is the main source of energy for systolic function. In a healthy heart, the oxidation of fatty acids, lactic acid, glucose, and ketone bodies provides about 50, 20, 15, and 10% of the total ATP, respectively (48). In the diabetic heart, the utilization of fatty acids and ketone bodies by cardiomyocytes increases, while the utilization of glucose decreases due to lipotoxicity and insulin resistance (49). The elevated peroxidation levels were detected *in vitro* which induced pluripotent stem cell-derived cardiomyocyte under the diabetic-like circumstance (50). Abnormal energy metabolism and accumulation of intermediate products affect the oxidative phosphorylation and TCA cycle, resulting in energy deficit. The kbhb-modified proteins enriched in these pathways might predict abnormal energy metabolism.

The functional enrichment analysis of downregulated kbhb proteins revealed that G3V885 (Myosin-6, Myh6), G3V8B0 (Myosin-7, Myh7), P11507 (sarcoplasmic/endoplasmic reticulum calcium ATPase 2), P16409 (myosin light chain 3), and P56741 (myosin-binding protein C) enriched in the pathogenic mechanism of dilated cardiomyopathy. It is reported that an autoimmune antibody against myosin is a risk factor for dilated cardiomyopathy which is defined by dilation and compromised contraction of the ventricle (51). Mutants in Myh7 might be attributed to the development of dilated cardiomyopathy (52). On the other hand, myosin also took part in the development of DCM. Howarth et al. found that the gene expression of Myh7 was upregulated, while Myh6 was downregulated in the heart of the type 2 Zucker diabetic fatty rat (53). The expression of Myh7 was decreased in the human-induced pluripotent stem cell-derived cardiomyocyte exposed to the diabetic-like environment (50). The earlier results show that dilated cardiomyopathy and DCM share some common pathogenic mechanisms, and more research studies are needed to uncover the hidden mechanism that might provide new insight into the diagnosis and treatment of DCM.

In summary, this study provided the first global investigation of the kbhb profile in DCM progression and proved that kbhb is related to many metabolic pathways and is mainly involved in energy metabolism. Our results could be an essential resource to explore DCM’s pathogenesis further. However, this study did not measure the βOHB in the urine and detected the localization of kbhb proteins in organelles. Further research is needed to comprehensively explore the role of kbhb in the development of DCM.

## Data availability statement

The original contributions presented in this study are publicly available. This data can be found here: http://proteomecentral.proteomexchange.org/cgi/GetDataset?ID=PXD038596, PXD038596.

## Ethics statement

The animal study was reviewed and approved by Ethics Review Committee of Zhengzhou University.

## Author contributions

WL, MH, and YL designed the research. WL, MH, and QL performed the experiments and analyzed the data. WL wrote the manuscript in consultation with MH and YL, who supervised the project. All authors discussed the results and contributed to the final manuscript.

## Footnotes

1
http://www.ebi.ac.uk/GOA/

2
http://meme-suite.org/tools/momo

3
https://david.ncifcrf.gov/tools.jsp

4
https://cn.string-db.org/

5
www.cytoscape.org
