# Supplementary material for: Proteome-wide analysis of lysine β-hydroxybutyrylation in the myocardium of diabetic rat model with cardiomyopathy
Source: Front Cardiovasc Med. 2023 Jan 9;9:1066822. doi: 10.3389/fcvm.2022.1066822 (PMC9868477; doi:10.3389/fcvm.2022.1066822)
Supplement: Supplementary file 2 [file Data_Sheet_1.pdf]

Supplementary data and figures

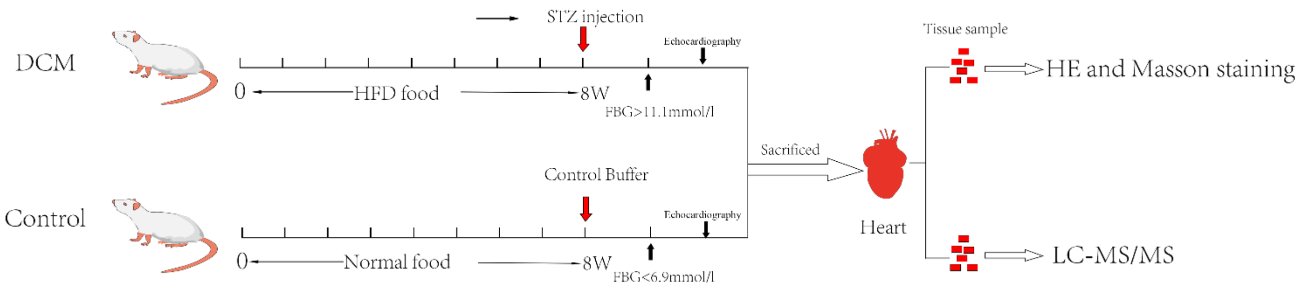

FIGURE 1

The work flow used in this study (n=3 rat per group).

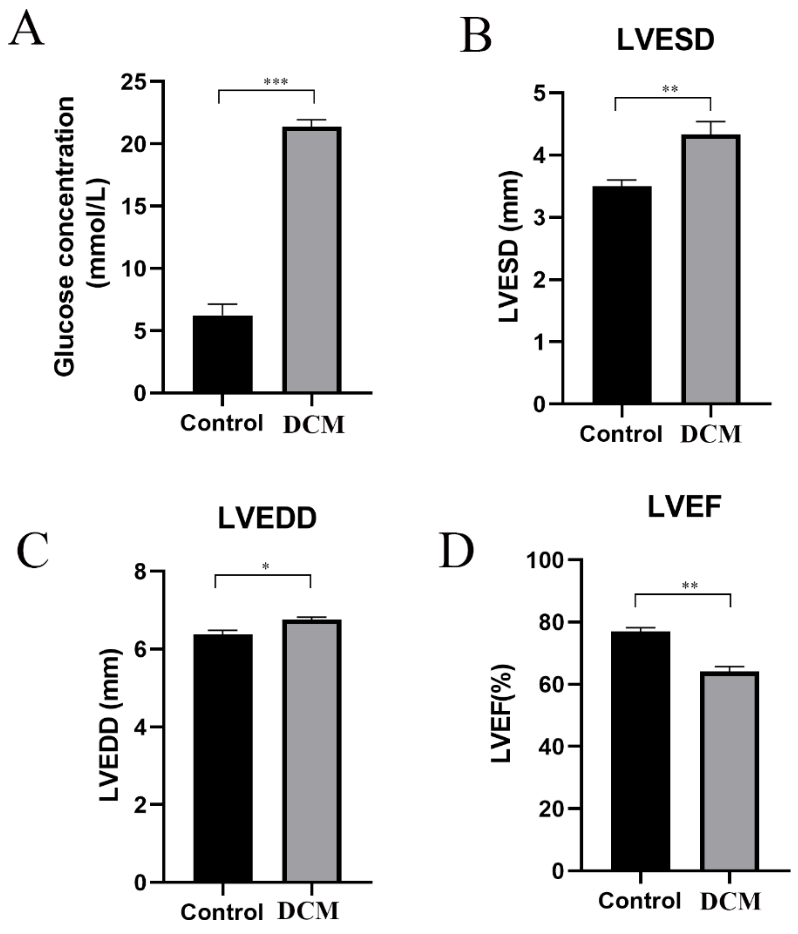

FIGURE 2

Establishment of diabetic cardiomyopathy rat modes. **(A)** The results of fasting blood glucose. **(B-D)** Cardiac function were evaluated by echocardiography (n=3 rat per group, $*P < 0.05$). LVESD = left ventricular end-systolic diameter; LVEDD = left ventricular end-diastolic diameter; LVEF = left ventricular ejection fraction. ** $p<0.01$

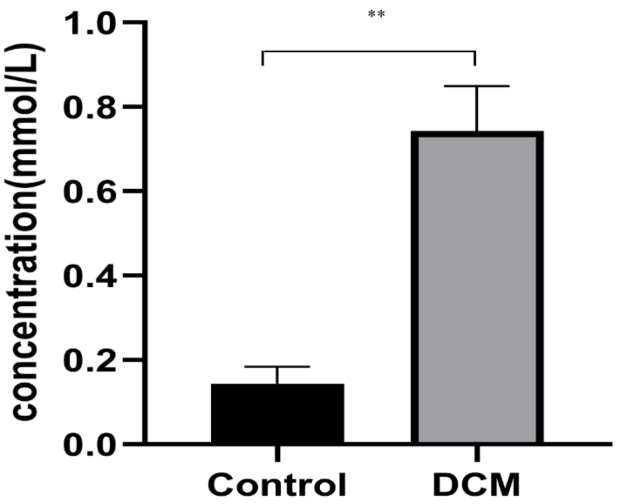

FIGURE 3
The concentration of BHB in the blood of control and DCM rats. n=3 rat per group. ** p<0.01

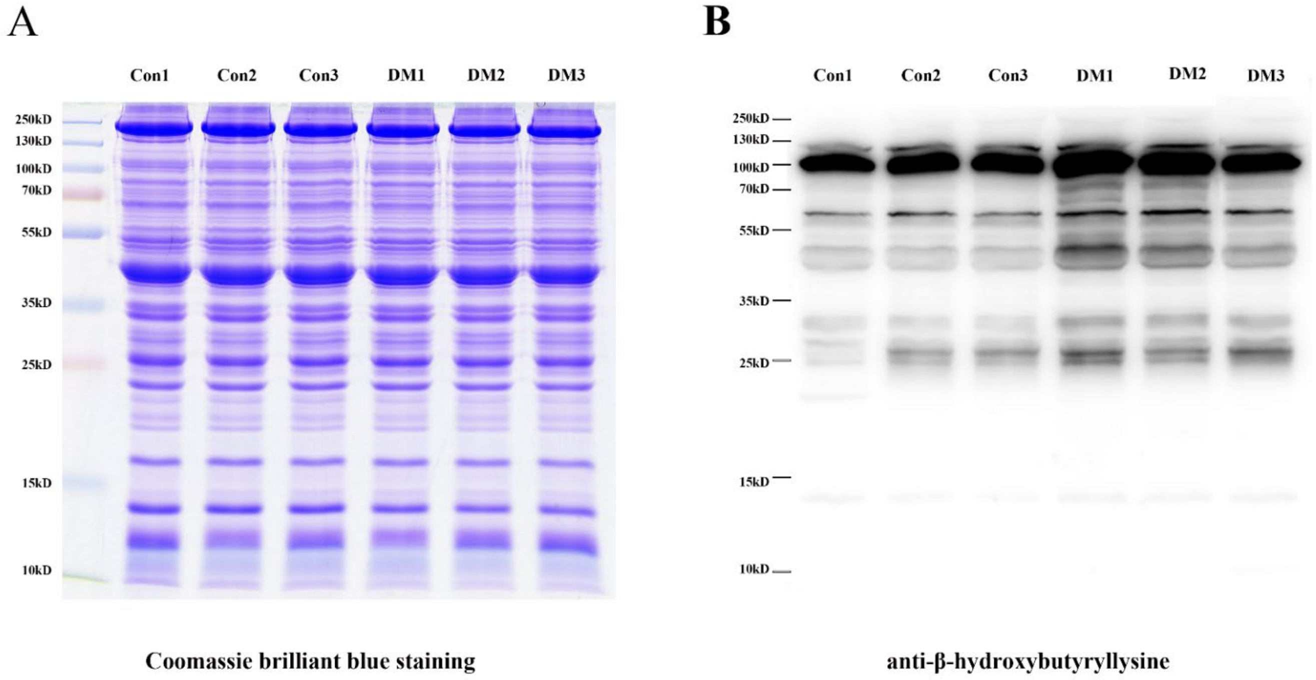

FIGURE 4
Detection of lysine β-hydroxybutyrylation in heart tissue of control and DM rats. **(A)** Coomassie brilliant blue staining **(B)** Western blot analysis of heart tissue lysates by anti-β-hydroxybutyryllysine antibody.

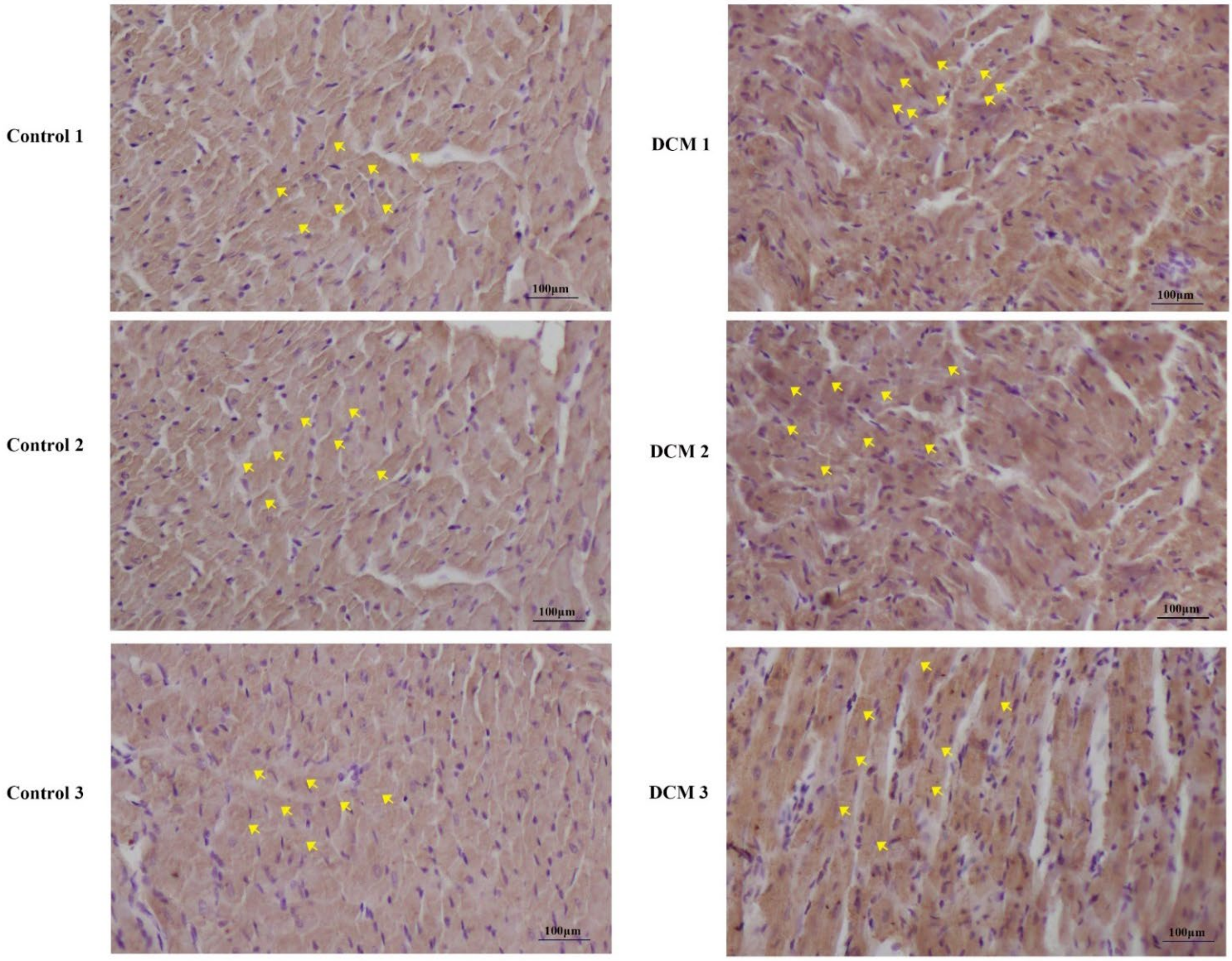

FIGURE 5
The β-hydroxybutyryl lysine expression in the control and DCM groups (Yellow arrows). Scale bar=100μm. n=3 rat per group, scale bar =100 μm

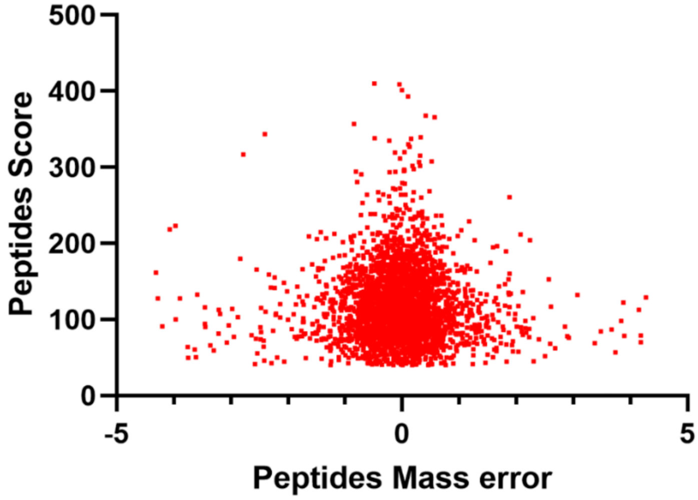

FIGURE 6
Distributions of mass errors for all the β-hydroxybutyryl peptides.
